# Extracellular Collagen VI Has Prosurvival and Autophagy Instructive Properties in Mouse Fibroblasts

**DOI:** 10.3389/fphys.2018.01129

**Published:** 2018-08-17

**Authors:** Silvia Castagnaro, Martina Chrisam, Matilde Cescon, Paola Braghetta, Paolo Grumati, Paolo Bonaldo

**Affiliations:** ^1^Department of Molecular Medicine, University of Padova, Padova, Italy; ^2^Institute of Biochemistry II, Goethe University, Frankfurt, Germany; ^3^CRIBI Biotechnology Center, University of Padova, Padova, Italy

**Keywords:** collagen VI, autophagy, mitophagy, apoptosis, fibroblasts, lysosomes

## Abstract

Collagen VI (ColVI) is an abundant and distinctive extracellular matrix protein secreted by fibroblasts in different tissues. Human diseases linked to mutations on ColVI genes are primarily affecting skeletal muscle due to non-cell autonomous myofiber defects. To date, it is not known whether and how fibroblast homeostasis is affected by ColVI deficiency, a critical missing information as this may strengthen the use of patients’ fibroblasts for preclinical purposes. Here, we established primary and immortalized fibroblast cultures from ColVI null (*Col6a1*^-/-^) mice, the animal model of ColVI-related diseases. We found that, under nutrient-stringent condition, lack of ColVI affects fibroblast survival, leading to increased apoptosis. Moreover, *Col6a1*^-/-^ fibroblasts display defects in the autophagy/lysosome machinery, with impaired clearance of autophagosomes and failure of Parkin-dependent mitophagy. *Col6a1*^-/-^ fibroblasts also show an increased activation of the Akt/mTOR pathway, compatible with the autophagy impairment, and adhesion onto purified ColVI elicits a major effect on the autophagic flux. Our findings reveal that ColVI ablation in fibroblasts impacts on autophagy regulation and cell survival, thus pointing at the new concept that this cell type may contribute to the pathological features of ColVI-related diseases.

## Introduction

The ECM microenvironment plays a crucial role for tissue homeostasis and ensures proper functionality through physical interactions and structural support. Several ECM components are involved in key cellular pathways other than the mere cell-matrix adhesion, such as activation and presentation of soluble factors, preservation of the stem cell niche, and signal transduction into cells (Hynes, 2009; Gattazzo et al., 2014). Recent studies also revealed that some ECM proteins are able to modulate macroautophagy (Neill et al., 2014). Macroautophagy (hereafter referred to as autophagy) is the degradative cellular pathway that involves the formation of double-membrane vesicles (autophagosomes) engulfing long-lived proteins, damaged organelles and pathogens, and delivering them to lysosomes for degradation and recycling (Boya et al., 2013). Extra- and intracellular nutrient and growth factor availability regulates autophagy by means of a complex machinery of interactors that enable a balanced adaptive response (Feng et al., 2014). Indeed, both defective and excessive activation of autophagy are detrimental for cell homeostasis and result in human degenerative pathologies (Levine and Kroemer, 2008; Schneider and Cuervo, 2014). The roles of the ECM on autophagy regulation are increasingly evident, although still under dissection (Neill et al., 2014; Buraschi et al., 2017).

Collagen VI is a major ECM protein forming a distinct microfibrillar network with a broad distribution in several embryonic and adult tissues (Cescon et al., 2015). It is composed of three major genetically distinct chains, α1(VI), α2(VI) and α3(VI), which associate intracellularly to form a triple helical monomer, followed by assembly into disulfide-bonded dimers and tetramers (Colombatti et al., 1995). After secretion in the extracellular space, tetramers associate in a non-covalent fashion forming beaded microfilaments, which are deposited in the ECM and interact with a number of other ECM and cell surface molecules (Kuo et al., 1997; Cescon et al., 2015). Mutations of ColVI genes in humans cause a range of diseases with variable onset and progression and primarily affecting skeletal muscles, including UCMD, BM and myosclerosis myopathy (Merlini et al., 2008b; Bönnemann, 2011; Bushby et al., 2014). A common and characteristic feature of these diseases is the involvement of joints, skin and connective tissue defects. The most characterized model of ColVI-related diseases is the ColVI null (*Col6a1*^-/-^) mouse, which displays an early onset myopathic phenotype with spontaneous apoptosis and accumulation of dysfunctional mitochondria within myofibers, due to defective regulation of autophagy (Irwin et al., 2003; Grumati et al., 2010). Similar defects were found in muscle biopsies of UCMD and BM patients (Angelin et al., 2007; Merlini et al., 2008a; Grumati et al., 2010), and following the demonstration that reactivation of autophagy by different means is beneficial in recovering the structural and functional defects of *Col6a1*^-/-^ mice, a pilot clinical trial in UCMD and BM patients by 1-year low-protein diet was successful in reactivating autophagy (Castagnaro et al., 2016). Although ColVI deficiency has a major impact on muscle fibers, the main producers of this protein in skeletal muscle, as in other tissues, are interstial fibroblasts (Braghetta et al., 2008; Zou et al., 2008). Fibroblast cultures from patients’ skin biopsies are extensively used to assess the effects of different mutations of ColVI genes in the synthesis, assembly, secretion and ECM deposition of the protein (Zhang et al., 2002; Pan et al., 2003; Jimenez-Mallebrera et al., 2006; Merlini et al., 2008a). Retention of mutated ColVI was observed in patients’ fibroblasts rather than in myofibers, suggesting that fibroblasts themselves may significantly contribute to the pathogenesis of ColVI diseases (Zou et al., 2008). However, no study until now investigated whether and how lack of ColVI impacts on the homeostasis and survival of fibroblasts.

In this work, we show that autophagy regulation is impaired in fibroblasts lacking ColVI. We generated immortalized fibroblasts from wild-type (WT) and *Col6a1*^-/-^ animals, and found that ColVI ablation triggers spontaneous apoptosis and defects in the autophagy-lysosome machinery of fibroblasts, together with a deregulation in the activity of the TFEB and of the mTOR signaling pathway. Moreover, we provide evidence that fibroblast adhesion onto purified native ColVI has instructive roles in promoting cell survival and autophagy, thus revealing a direct regulation of autophagy by extracellular ColVI.

## Materials and Methods

### Cell Culture and Transfection

Primary murine embryonic fibroblasts (MEF) were prepared by dissociation of E13.5 embryos generated from WT and *Col6a1*^-/-^ mice in the C57BL/6N background (Irwin et al., 2003; Grumati et al., 2010). Animal procedures were approved by the Ethics Committee of the University of Padova, in accordance to EU Directive for animal experiments, and authorized by the Italian Ministry of Health. MEF were cultured in Dulbecco’s modified Eagle’s medium (DMEM; Gibco) supplemented with 10% fetal bovine serum (FBS; Life Technologies), 0.2 M L-glutamine and 1:100 penicillin-streptomycin (Life Technologies). Early passage MEF were immortalized using a pMSE plasmid encoding for SV40 large T-antigen (SV40LT, kindly provided by Dr. M. Sandri, University of Padova) by microporation (Neon^®^ Transfection System, MPT100, Life Technologies). MEF lines stably expressing GFP-LC3 were obtained from E12 embryos generated from GFP-LC3 mice (Mizushima et al., 2004) (provided by Riken BRC; GFP-LC3#53 strain, RBRC00806) and GFP-LC3::*Col6a1^-/-^* mice (Chrisam et al., 2015). For all the experiments, MEF were cultured in DMEM supplemented with 10% FBS (complete medium) and maintained in a humidified incubator containing 5% CO_2_ at 37°C, and periodically tested for contamination with LookOut^®^ Mycoplasma PCR Detection Kit (Sigma-Aldrich). For ColVI expression studies, cells were cultured 3–7 days in complete medium supplemented with 0.25 mM ascorbic acid. When indicated, cells were plated on plastic dishes coated with 5 μg/cm^2^ purified murine native ColVI (Irwin et al., 2003) or with collagen I (C8919, Sigma-Aldrich), and cultured for 3 days. For transfection experiments, 0.8 × 10^5^ cells were plated on 12 mm glass coverslips, grown to 80% confluence and transfected with pMitoRed (kindly provided by Dr. L. Scorrano, University of Padova), mCherry-Parkin (Addgene plasmid 23956), YFP-Parkin (Addgene plasmid 23955; Narendra et al., 2008) or GFP-TFEB (kindly provided by Dr. M. Sandri, University of Padova) plasmids using Lipofectamine^TM^ LTX (Life Technologies), according to manufacturer’s guidelines. After 4 h, transfected cells were washed in complete DMEM without antibiotics and cultured 18–20 h before fixation with 4% paraformaldehyde in phosphate-buffered saline (PBS). When indicated, the following additional treatments were used on subconfluent cells (about 80% confluence): 3 or 6 h serum withdrawal; 50 μM CQ (Sigma-Aldrich); 250–500 μM 3-MA (Sigma-Aldrich); 20 μM CCCP (Sigma-Aldrich). Finally, cells were harvested and processed for flow cytometry analyses or fixed for 10 min with ice-cold acetone/methanol (1:1) at -20°C.

### Immunofluorescence on Cells

Fixed cells were washed three times in PBS, treated for 30 min with 10% goat serum (Sigma-Aldrich) and incubated for 2 h at room temperature or overnight at 4°C with the following primary antibodies: rabbit anti-ColVI (AS72, kindly supplied by Dr. A. Colombatti, CRO Aviano), mouse anti-fibronectin (kindly supplied by Dr. A. Colombatti), rabbit anti-α1(VI) (Santa Cruz Biotechnology), guinea pig anti-α3(VI) (kindly supplied by Dr. R. Wagener, University of Cologne), rat anti-LAMP-2 (Abcam), rabbit anti-LC3 (Cell Signaling Technologies), rabbit anti-Tom20 (Santa Cruz Biotechnology). After three washing in PBS, slides were incubated for 1 h with the following secondary antibodies: anti-mouse Cy2, anti-rabbit Cy2, anti-rabbit Cy3, anti-rat Cy3 (all Jackson Immunoresearch); IRIS anti-rabbit Cy5.5 (Cyanine Technologies). Nuclei were stained with Hoechst 33258 (Sigma-Aldrich). Slides were mounted in 80% glycerol-PBS and analyzed with a Leica SP5 confocal microscope. Co-localization was quantified in merged images, after thresholding of individual frames, using the JACoP plugin of the ImageJ software. The percentage of cells with enlarged lysosomes was calculated by manual counting of the cells with at least two enlarged LAMP-2-positive lysosomes on total cell number per field, using thirty randomly chosen image fields. The percentage of cells with tubular or fragmented mitochondria was estimated by manual analysis of mitochondrial morphology in at least thirty randomly chosen fields. Mean data are representative of at least five independent biological samples.

### TUNEL Assay

Terminal deoxynucleotidyl transferase dUTP-mediated nick-end labeling (TUNEL) analysis was performed on acetone/methanol fixed MEF using the Dead End Fluorometric *In situ* Apoptosis Detection System (Promega). Cells, grown on slides and fixed in acetone/methanol, were washed and incubated for 10 min with the equilibration buffer. Then the slides were incubated with a buffer containing fluorescent nucleotides, terminal deoxynucleotidyl transferase (TdT), and Hoechst 33258 (Sigma-Aldrich) for 1 h at 37°C. The reaction was blocked with SSC solution (300 mM NaCl, 30 mM sodium citrate). After being washed three times in PBS, slides were mounted using 80% glycerol. Hoechst was used to counterstain all nuclei and TUNEL-positive nuclei were determined by counting randomly selected fields using a Zeiss Axioplan microscope.

### Flow Cytometry

Wild-type and *Col6a1*^-/-^ MEF were plated (200,000 cells/well) in 12-well plates by adhesion onto either plastic, purified ColVI or collagen I, and cultured for 24 h. Cells were washed in PBS, and incubated for 3 or 6 h in complete medium or in serum-free medium. When indicated, 1 μM staurosporine or 250–500 μM 3-MA (Sigma-Aldrich) were added to the serum-free medium. Apoptosis was determined using the Annexin V (AnV)-FITC Apoptosis detection kit (eBioscience). Cells were harvested, washed with PBS and incubated in 195 μl binding buffer containing 5 μl AnV-FITC for 15 min at room temperature in the dark, according to the manufacturer’s protocol. Cells were finally incubated with 1 μg/μl PI and samples were immediately analyzed on a FACSCanto flow cytometer equipped with FACS Diva software (BD Biosciences), using FL-1 and FL-2 settings. Forward scatter and side scatter (morphology parameters) were performed to discriminate live cells (AnV-negative and PI-negative), early apoptotic cells (AnV-positive and PI-negative), late apoptotic cells (AnV-positive and PI-positive), and AnV-negative and PI-positive cells (mainly necrotic). For each sample 10,000 events were collected from five independent biological replicates. Results were analyzed using Flowing Software v2.5.0 (Turku Centre for Biotechnology, University of Turku).

### Quantitative Real-Time PCR (qRT-PCR)

500,000 MEF of each genotype were plated in 6-well plates, in triplicate, and cultured for 2 days (autophagy studies) or 4 days (ColVI expression). For the analysis of ColVI genes, cells were cultured until post-confluence in DMEM containing 10% FBS, in the presence or absence of 0.25 mM ascorbic acid. For the analysis of autophagy genes, cells were washed in PBS, then cultured for 3 h in medium with or without FBS. RNA extraction was performed by adding 1 ml/well TRIzol Reagent (Life Technologies) directly on cultured cells and following the manufacturer’s protocol. RNA was quantified using a Nanodrop ND-1000 instrument (Nanodrop Technologies) and 1 μg total RNA was retrotranscribed using the SuperScript III First-Strand Synthesis System for RT-PCR (Life Technologies), following manufacturer’s instructions. Resulting cDNAs were used to perform quantitative real time PCR using Rotor-Gene SYBR Green PCR Kit mastermix (Qiagen) with the RotorGeneQ instrument from Qiagen. Primer sequences are provided in **Supplementary Table S1**.

### Western Blotting

Mouse embryonic fibroblasts (0.5 × 10^6^ cells/ ml) were cultured in 6-well plates for 2 days. When indicated, multiwell plates were coated with 5 μg/cm^2^ native ColVI or collagen I as described above. Cells were washed once in PBS and harvested with a cell lifter in lysis buffer (50 mM Tris-HCl, pH 7.5, 150 mM NaCl, 20 mM EDTA, 0.5% NP40), and supplemented with phosphatase inhibitors (Cocktail II P5726, Sigma-Aldrich) and proteases inhibitors (Complete EDTA-free, Roche). Cell lysates were then sonicated twice for 15 s using a Bioruptor (Diagenode) and the cleared cell lysates were quantified with the BCA Protein Assay kit (Pierce). SDS-PAGE of protein lysates (20 or 30 μg) was performed under reducing condition, using 3–8%, 4–12%, 10%, or 12% polyacrylamide Novex NuPAGE Bis-Tris gels (Life Technologies) and electrotransferred onto PVDF membrane (Merck Millipore). Membranes were blocked for 1 h in 5% milk in Tris-buffered saline, 0.1% Tween 20 (Sigma-Aldrich) and incubated 1 h at room temperature or overnight at 4°C with the primary antibodies diluted 1:1000 in TBST with 5% BSA. For a complete list of the antibodies, see **Supplementary Table S2**. Membranes were then washed three times and incubated for 1 h at room temperature with horseradish peroxidase-conjugated secondary antibodies (Amersham BioSciences) diluted 1:1000 in TBST and 5% milk. Detection was performed by SuperSignal West Pico or Dura Chemiluminescent Substrate and CL-X Posure Film (Thermo Scientific), using β-actin as a loading control. When needed, membranes were stripped using a stripping buffer (25 mM glycine, 1% SDS, pH 2.0) and re-probed. Western blots were performed in at least three independent experiments. Densitometric measurements were obtained with the ImageJ software, by normalizing the signal of each band to the corresponding β-actin band or to the non-phosphorylated form of protein. When indicated, each band was normalized to the first lane (arbitrarily set as 1) of the relative gel.

### Statistical Analysis

All results are expressed as means ± SEM. Statistical analyses were performed by one way ANOVA with *post hoc* Bonferroni-correction where appropriate, as indicated in figure legends, or by Student’s *t*-test for unpaired data. GraphPad Prism software was used, and *P* < 0.05 was considered statistically significant.

## Results

### Immortalized Mouse Fibroblasts Secrete ColVI and Organize an ECM

We established stable fibroblast cultures from WT and *Col6a1*^-/-^ mice, and analyzed by different approaches the production of ColVI and other ECM proteins. Immunofluorescence showed the presence of organized ColVI filaments in the extracellular space of WT fibroblasts, whereas labeling was negative in *Col6a1*^-/-^ cultures as expected (**Supplementary Figure S1A**). Fibronectin was detected in the ECM of both WT and *Col6a1*^-/-^ fibroblasts, and as previously reported (Tillet et al., 1994) its labeling was strictly interconnected to ColVI staining (**Supplementary Figures S1A,B**). Western blotting confirmed the presence of ColVI chains in cell extracts and culture media of WT fibroblasts, and ascorbic acid promoted secretion of the protein in WT cultures (**Supplementary Figure S1C**). In the absence of α1(VI) chain, the intracellular protein levels of the other two chains were strongly decreased and ColVI secretion was abolished in *Col6a1*^-/-^ fibroblasts (**Supplementary Figure S1C**). qRT-PCR analysis showed that WT fibroblasts express high levels of *Col6a1*, *Col6a2* and *Col6a3* transcripts (**Supplementary Figure S1D**). In *Col6a1*^-/-^ fibroblasts, *Col6a1* transcripts were undetectable as expected, whereas *Col6a3* transcript levels were markedly decreased and *Col6a2* mRNA levels were similar to WT fibroblasts (**Supplementary Figure S1D**). Given the presence of low but discrete amounts of intracellular α2(VI) and α3(VI) polypeptides in *Col6a1*^-/-^ fibroblasts, we assessed whether the non-secreted ColVI chains were degraded through the autophagic pathway. This analysis did not reveal any co-localization of intracellular α3(VI) with autophagosome dots, suggesting that the unassembled ColVI chains are not degraded via the autophagy-lysosome pathway (**Supplementary Figures S1E,F**).

### ColVI Ablation Triggers Fibroblast Apoptosis via Caspase Activation

**FIGURE 1:**
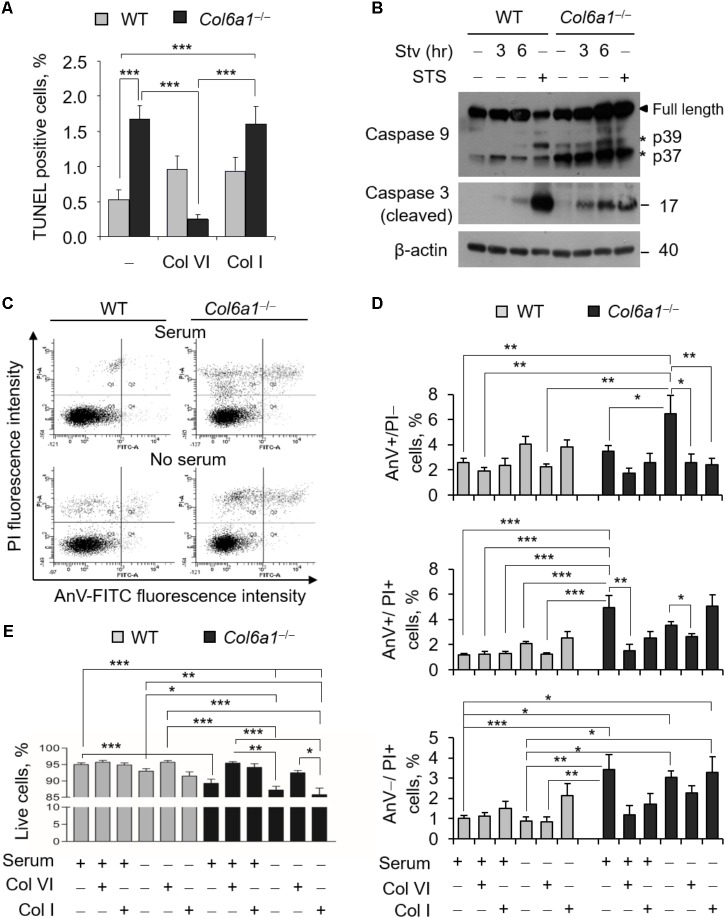
Apoptosis is increased in *Col6a1*^-/-^ fibroblasts and adhesion onto ColVI rescues the phenotype. **(A)** Quantification of TUNEL-positive nuclei in WT and *Col6a1*^-/-^ fibroblasts cultured for 2 days on plastic wells (–), or on wells coated with purified ColVI (Col VI) or collagen I (Col I) proteins. **(B)** Western blot analysis of total cell extracts from WT and *Col6a1*^-/-^ fibroblasts cultured in complete DMEM with 10% serum, or following serum starvation for 3 and 6 h. Where indicated, cells were treated with 1 μM staurosporine for positive control of caspase activation. **(C–E)** Flow cytometry analysis for AnV and PI in WT and *Col6a1*^-/-^ fibroblasts cultured for 2 days on plastic, ColVI (Col VI), or collagen I (Col I), and serum depleted for the final 3 h where indicated. Data indicate the average for 10,000 cells collected in at least five independent biological samples. **(C)** Representative dot plots of flow cytometry analysis for AnV and PI. **(D)** Histograms of the quantification of cellular events obtained by flow cytometry, as shown in C. Percentages of AnV-positive/PI-negative (early apoptotic cells, top panel), AnV-positive/PI-positive (late apoptotic cells, middle panel), and AnV-negative/PI-positive cells (bottom panel) are shown. **(E)** Quantification of the percentage of live cells (AnV- and PI-negative cells) in the different conditions. Data represents the mean of at least five independent experiments, and were analyzed by ANOVA test with Bonferroni correction. ^∗^*P* < 0.05; ^∗∗^*P* < 0.01; ^∗∗∗^*P* < 0.001. AnV, annexin V; PI, propidium iodide; STS, staurosporine; Stv, serum starvation.

To evaluate the incidence of apoptosis in WT and *Col6a1*^-/-^ fibroblasts, we first performed TUNEL analysis. Apoptotic nuclei were markedly increased in ColVI null fibroblasts (**Figure 1A**). Notably, apoptosis was significantly decreased in *Col6a1*^-/-^ fibroblasts following adhesion onto purified ColVI for 2 days, whereas adhesion on collagen type I, used as a control ECM substrate, did not display the same effect (**Figure 1A**). Western blotting showed increased levels of cleaved caspases 3 and 9 in *Col6a1*^-/-^ fibroblasts compared to WT ones, both in standard conditions and following serum starvation for 3 or 6 h (**Figure 1B** and **Supplementary Figure S2**). We further assessed cell death by flow cytometry, following cell labeling with AnV and PI. PI staining allows to discriminate between early apoptotic/healthy cells, having an intact membrane, and late stage apoptotic/necrotic cells, in which compromised membranes allow the incorporation of the dye. Flow cytometry confirmed that apoptosis incidence was increased in *Col6a1*^-/-^ fibroblasts with respect to WT ones, particularly after serum depletion (**Figures 1C,D**). Indeed, the percentage of AnV-positive/PI-negative (early apoptotic) cells was increased in *Col6a1*^-/-^ fibroblasts after serum depletion, and adhesion onto ColVI decreased the number of early apoptotic cells in these cultures (**Figure 1D**, upper panel). The percentage of AnV-positive/PI-positive (late apoptotic) cells was higher in *Col6a1*^-/-^ fibroblasts than in WT cells, and adhesion onto ColVI rescued late apoptotic events both in standard conditions and following serum withdrawal (**Figure 1D**, middle panel). In addition, the percentage of AnV-negative/PI-positive cells was also significantly increased in *Col6a1*^-/-^ cultures, both in complete medium and after serum starvation (**Figure 1D**, lower panel). Collectively, when evaluating live cells, *Col6a1*^-/-^ fibroblasts were less viable when compared to WT fibroblasts, both under standard conditions and after serum depletion for 3 h (**Figure 1E**) or 6 h (data not shown). Interestingly, 2-day culture onto purified ColVI decreased the incidence of apoptosis in *Col6a1*^-/-^ cultures both under standard conditions and after serum withdrawal, thus improving the survival of *Col6a1*^-/-^ fibroblasts in both conditions (**Figure 1E**). By contrast, when grown onto a control coating made of collagen I, *Col6a1*^-/-^ fibroblasts did not rescue cell viability after serum withdrawal (**Figures 1D,E**).

### The Autophagic Flux Is Impaired in *Col6a1*^-/-^ Fibroblasts

**FIGURE 2:**
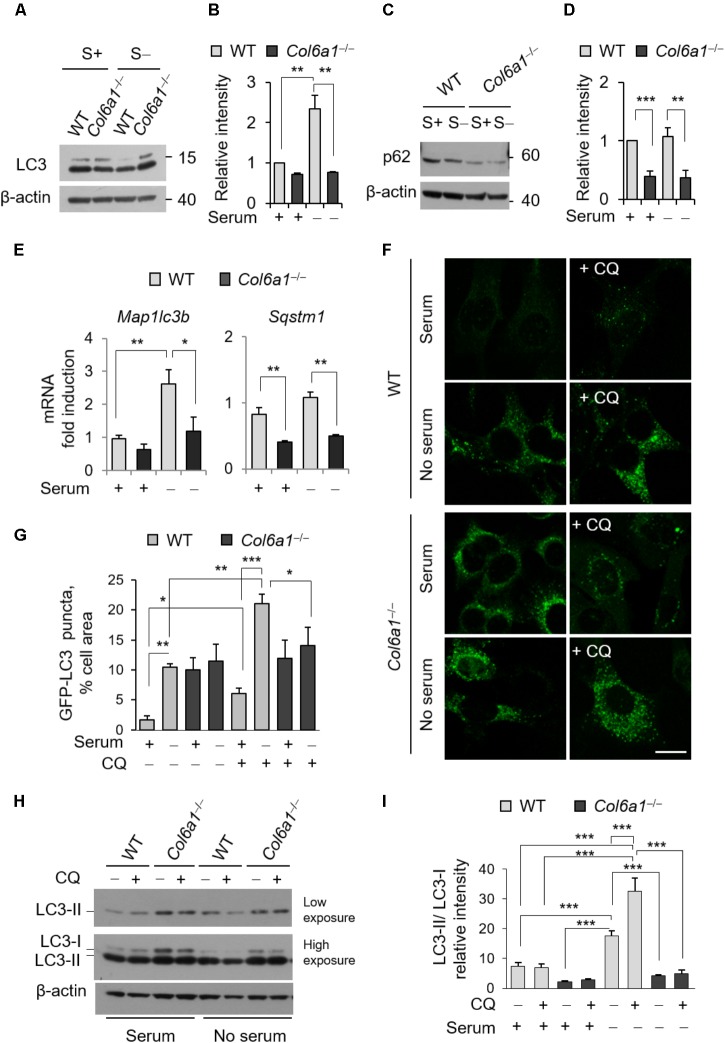
The autophagic flux is impaired in *Col6a1*^-/-^ fibroblasts. **(A–D)** Western blot analysis of LC3 and p62 in total cell extracts from WT and *Col6a1*^-/-^ fibroblasts in complete medium (S+) or following serum withdrawal for 3 h (S–). **(B,D)** Show respectively the LC3-II/LC3-I ratio **(B)** and the p62/β-actin ratio **(D)**, as determined by densitometric quantification of the western blots as in **(A,C)**. Values for WT fibroblasts in complete medium were arbitrarily set to 1. Data represents the mean of at least three independent experiments. **(E)** qRT-PCR analysis of *Map1lc3b* and *Sqstm1* mRNA levels. Data represents the mean of at least three independent experiments. **(F)** Fluorescence microscopy detection of LC3-positive puncta in WT and *Col6a1*^-/-^ fibroblasts derived from *Col6a1*^+/+^::GFP-LC3 and *Col6a1*^-/-^::GFP-LC3 mice, respectively, and maintained in complete medium or subjected to serum starvation for 3 h. LC3 puncta (autophagosomes) accumulate in *Col6a1*^-/-^ fibroblasts both in complete medium and serum starved conditions. Scale bar, 25 μm. **(G)** Quantification of GFP-LC3 puncta per cell area, as shown in **(F)**. **(H)** Western blot analysis of total cell extracts from WT and *Col6a1*^-/-^ fibroblasts in complete medium (S+) or following serum withdrawal for 3 h (S–). The autophagic flux, as determined by the analysis of LC3 lipidation in the absence or presence of 50 μM CQ treatment, is shown. **(I)** Densitometric quantification of the relative intensity of LC3-II/LC3-I ratio of three independent western blot experiments, as in **(H)**. Data were analyzed by ANOVA test with Bonferroni correction. ^∗^*P* < 0.05; ^∗∗^*P* < 0.01; ^∗∗∗^*P* < 0.001.

To examine whether autophagy was affected in *Col6a1*^-/-^ fibroblasts, we first investigated autophagosome formation, by evaluating the conversion of cytosolic MAP1LC3B (LC3-I) to its lipidated form (LC3-II), as well as autophagy-dependent protein degradation, by monitoring protein level of the specific cargo receptor Sqstm1/p62 protein, both in complete medium and after 3 h serum withdrawal. Western blotting showed that the LC3-II/LC3-I ratio was decreased in *Col6a1*^-/-^ fibroblasts after serum withdrawal (**Figures 2A,B**). In addition, p62 protein levels were decreased in *Col6a1*^-/-^ fibroblasts with respect to WT, both under basal conditions and after serum withdrawal (**Figures 2C,D**). Gene expression analysis by qRT-PCR showed that the transcriptional activation of LC3 was defective in ColVI deficient fibroblasts, as we did not detect any significant increase of *Map1lc3b* mRNA levels in *Col6a1*^-/-^ cultures after serum starvation, at difference from WT ones (**Figure 2E**). *Sqstm1* transcript levels were down-regulated in *Col6a1*^-/-^ fibroblasts, in agreement with the decrease of p62 protein levels and independently of serum starvation (**Figure 2E**).

We also monitored autophagosome formation by detection of GFP-positive puncta in fibroblasts derived from *Col6a1*^+/+^::GFP-LC3 and *Col6a1*^-/-^::GFP-LC3 reporter mice (**Figure 2F**). *Col6a1*^+/+^::GFP-LC3 fibroblasts displayed few GFP-positive puncta in complete medium, while 3 hr serum starvation elicited a sixfold increase of GFP-positive puncta (**Figure 2G**). To investigate the dynamics of the autophagic flux we used CQ, an inhibitor of lysosome acidification. Treatment of *Col6a1*^+/+^::GFP-LC3 fibroblasts with 50 μM CQ led to a marked accumulation of autophagosomes both in basal conditions and upon serum starvation, showing an increase in the autophagy flux upon serum withdrawal as expected (**Figures 2F,G**). In contrast, *Col6a1*^-/-^::GFP-LC3 fibroblasts displayed massive accumulation of GFP-positive puncta already in complete medium condition, without any substantial variation in the number of fluorescent puncta after serum starvation or following CQ treatment (**Figures 2F,G**). Consistently, western blotting showed that 3 h serum starvation elicited a marked increase of the LC3-II/LC3-I ratio in WT fibroblasts both in the absence and in the presence of CQ (two- and four-fold increase, respectively), indicating increased autophagy induction (**Figures 2H,I**). In particular, LC3-II/LC3-I ratio of WT fibroblasts was increased when CQ was added to serum starved cultures, pointing at the extensive formation of autophagosomes in response to starvation. Differently, *Col6a1*^-/-^ fibroblasts did not displayed any significant increase of the LC3-II/LC3-I ratio after serum starvation (which was significantly decreased with respect to WT cells) and no further increase after CQ treatment (**Figures 2H,I**).

The presence of accumulated autophagosomes in *Col6a1*^-/-^ fibroblasts, together with the increased spontaneous apoptosis (**Figure 1**), prompted the possibility that an autophagy-dependent cell death may occur in ColVI deficient fibroblasts. To test this hypothesis, we investigated the incidence of AnV- and PI-positive cells in the absence or presence of the autophagy inhibitor 3-MA. Treatment with 3-MA for 3 h led to a dose-dependent increase of cell death both in WT and *Col6a1*^-/-^ fibroblasts (**Supplementary Figure S3**), thus excluding the possibility of a substantial influence of autophagy-dependent cell death in ColVI deficient fibroblasts.

### ColVI Ablation Affects Lysosomes and Impairs Autophagosome-Lysosome Fusion

**FIGURE 3:**
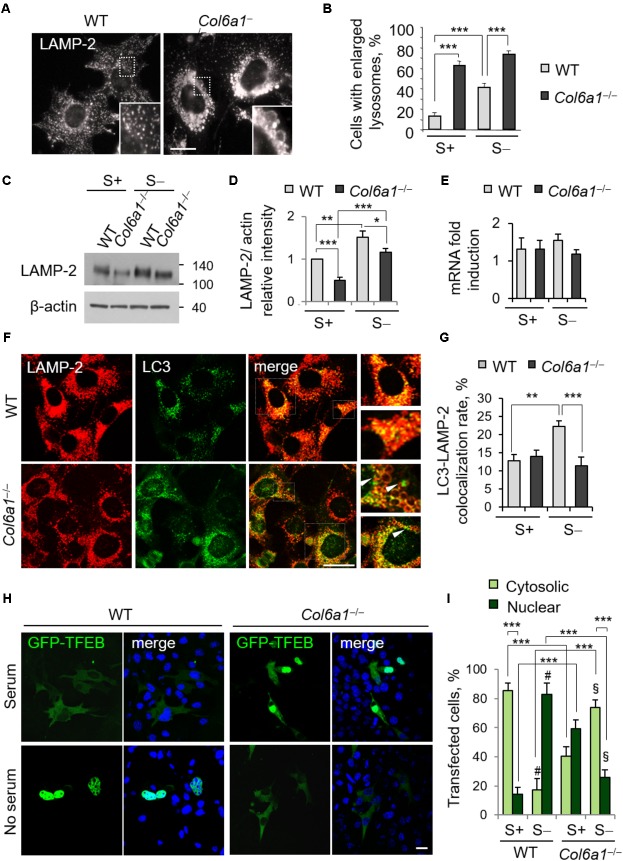
Collagen VI ablation leads to defects in autolysosome formation and TFEB translocation. **(A)** Immunofluorescence for LAMP-2, showing normal lysosomes in WT fibroblasts and enlarged lysosomes in *Col6a1*^-/-^ fibroblasts. The insets show a magnification of the respective boxed areas. Scale bar, 25 μm. **(B)** Quantification of cells containing at least two enlarged lysosomes. Data represent the mean percentages ± SEM of cells with enlarged lysosomes, ad determined from thirty images per condition. **(C)** Western blot analysis of LAMP-2 in WT and *Col6a1*^-/-^ fibroblasts, in complete medium (S+) or following serum withdrawal for 3 h (S–). **(D)** Densitometric quantification of the relative LAMP-2/β-actin ratio of three independent western blot experiments, as in **(C)**. The value for WT fibroblasts in complete medium was arbitrarily set to 1. **(E)** qRT-PCR analysis of *Lamp2* mRNA levels. **(F)** Co-immunostaining for LAMP-2 and LC3 in WT and *Col6a1*^-/-^ fibroblasts, following 3 h serum withdrawal. The right panels show a higher magnification of the boxed area of the respective merged image. *Col6a1*^-/-^ fibroblasts display impaired autophagosome (green puncta) fusion with lysosomes (red) (arrowheads). Scale bar, 25 μm. **(G)** Co-localization rate of LC3 and LAMP-2 staining in complete medium (S+) or following serum withdrawal for 3 h (S–). Mean percentages ± SEM were calculated for at least fifteen images per condition. **(H)** Representative fluorescence microscopy images of WT and *Col6a1*^-/-^ fibroblasts in complete medium and after 3 h serum withdrawal, following transfection with a GFP-TFEB expression construct (green). Nuclei were stained with Hoechst (blue). Scale bar, 25 μm. **(I)** Quantification of transfected WT and *Col6a1*^-/-^ fibroblasts showing either cytosolic or nuclear GFP-TFEB, as shown in **(G)**. Data represents the mean of at least three independent experiments, and were analyzed by ANOVA test with Bonferroni correction. ^∗^*P* < 0.05; ^∗∗^*P* < 0.001; ^∗∗∗^*P* < 0.0001; ^#^*P* < 0.001 for the comparison between WT complete medium and WT no serum; ^§^ *P* < 0.001 for the comparison between *Col6a1*^-/-^ complete medium and *Col6a1*^-/-^ no serum. S+, complete medium; S–, 3 h serum withdrawal.

The finding that lack of ColVI causes autophagosome accumulation pointed at an impairment of the autophagy-lysosome machinery in *Col6a1*^-/-^ fibroblasts, with a defective “off-rate” of the autophagic flux. Thus, we analyzed lysosomes and their fusion with autophagosomes. Immunostaining with the lysosomal marker LAMP-2 revealed high amounts of enlarged lysosomes in *Col6a1*^-/-^ fibroblasts, whereas in WT cultures lysosomes appeared as smaller punctuate structures (**Figure 3A**). Enlarged lysosomes were only detectable in WT fibroblasts after serum depletion (albeit to a lesser extent when compared to *Col6a1*^-/-^ cells) or following CQ treatment (**Figure 3B** and **Supplementary Figure S4**). Moreover, the increased number of enlarged lysosomes in *Col6a1*^-/-^ fibroblasts appeared to be independent on serum starvation (**Figure 3B**). Interestingly, when compared to WT, *Col6a1*^-/-^ fibroblasts also displayed a decrease in LAMP-2 protein levels (**Figures 3C,D**), which was not paralleled by a decrease of *Lamp2* mRNA levels (**Figure 3E**). We further analyzed the final stages of fusion between autophagosomes and lysosomes, by performing co-immunostaining for LC3 and LAMP-2 (**Figure 3F** and **Supplementary Figure S5**). In WT cultures, co-localization of autophagosomes with lysosomes (fusion events) was almost doubled upon serum depletion-induced autophagy, whereas in *Col6a1*^-/-^ cultures the co-localization of LAMP-2 and LC3 was not affected by serum depletion (**Figure 3G**), indicating defective autophagosome-lysosome fusion. Analysis of *Col6a1*^+/+^::GFP-LC3 and *Col6a1^-/-^*::GFP-LC3 fibroblasts provided similar results (**Supplementary Figure S6**).

A proper cross-talk between autophagosomes and lysosomes is fundamental to ensure an efficient completion of the autophagy process. The accumulation of autophagosomes and the lysosomal abnormalities prompted us to investigate whether the cellular clearance via lysosomes was affected in *Col6a1^-/-^* fibroblasts. One of the master regulators of lysosomal function and autophagy is the TFEB. The extracellular signal-regulated kinase 2 (Erk2) and mTOR coordinate TFEB retention in the cytosol, thus regulating its activity. Under nutrient depletion, TFEB rapidly shuttles to the nucleus and activates its translational program (Settembre et al., 2011; Russell et al., 2014). Hence, we investigated TFEB cellular localization following transfection with a GFP-TFEB construct. As expected, in WT fibroblasts serum depletion induced TFEB translocation from the cytosol to the nucleus, and indeed the majority of cells showed GFP-TFEB in the nucleus after 3 h serum depletion (from 14% in complete medium, to 82% in serum starvation; **Figures 3H,I**). Conversely, in *Col6a1*^-/-^ fibroblasts the nutrient availability-dependent cellular localization of GFP-TFEB was strongly affected. Indeed, in complete medium, 59% of *Col6a1*^-/-^ cells displayed GFP-TFEB already in the nucleus, whereas after 3 h serum depletion nuclear translocation of GFP-TFEB was markedly reduced, being present in 26% of cells) (**Figures 3H,I**).

### *Col6a1*^-/-^ Fibroblasts Display Altered Activity of Autophagy Regulatory Signals and of the mTOR Pathway

**FIGURE 4:**
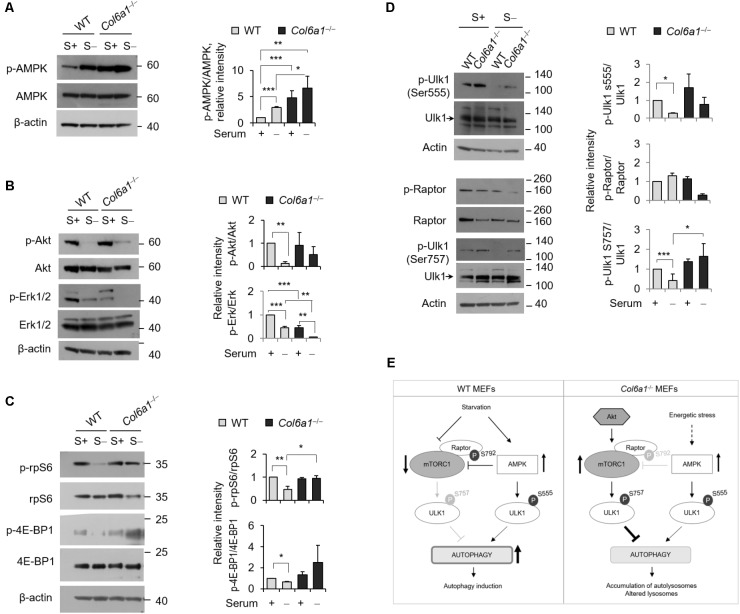
AMP-activated protein kinase (AMPK) and mTOR signaling are hyperactivated in *Col6a1*^-/-^ fibroblasts. Western blotting of total cell extracts from WT and *Col6a1*^-/-^ fibroblasts in complete medium (S+) or following serum withdrawal for 3 h (S–). **(A)** Analysis of AMPK activation (left) and densitometric quantification of phospho-AMPK vs. total AMPK (right). **(B)** Phosphorylation of Akt and Erk1/2. Densitometric quantifications of the phosphorylated forms of phospho-Akt vs. total Akt and of phospho-Erk vs. total Erk are shown on the right panels. **(C)** Analysis of the mTOR downstream targets 4E-BP1 and rpS6. Densitometric quantifications of the phospho-rpS6 vs. rpS6 and of the phospho-4E-BP1 vs. 4E-BP1 are shown on the right panels. **(D)** Phosphorylation of Ulk1 (Ser555), Raptor (Ser792), and Ulk1 (Ser757). Densitometric quantifications of phospho-Raptor *vs* total Raptor and of phospho-Ulk1 vs. total Ulk1 are shown on the right panels. For all the densitometric quantification, data represents the mean of at least three independent western blots experiments, and values for WT fibroblasts in complete medium were arbitrarily set to 1. ^∗^*P* < 0.05; ^∗∗^*P* < 0.001; ^∗∗∗^*P* < 0.0001. **(E)** Schematic diagram of AMPK and mTOR signaling and their impact on autophagy regulation in WT and *Col6a1*^-/-^ fibroblasts.

We analyzed the activation of AMPK and found that the levels of phosphorylated AMPK were increased in *Col6a1*^-/-^ cultures, with respect to WT ones (**Figure 4A**). Analysis of the activation status of Akt and of Erk1/2, two kinases acting on autophagy, showed that Akt phosphorylation was slightly enhanced in *Col6a1*^-/-^ cultures after serum starvation, whereas both Erk1/2 kinases were much less phosphorylated in *Col6a1*^-/-^ cultures compared to WT ones, both in basal conditions and after serum starvation (**Figure 4B**). Furthermore, the mTOR axis was persistently activated in *Col6a1*^-/-^ fibroblasts after serum deprivation, as revealed by phosphorylation of 4E-BP1 and of the S6 ribosomal protein (rpS6), two major downstream targets of mTORC1 (**Figure 4C**). To assess the coordinated activity of AMPK and of Akt/mTOR axis in these cultures, we analyzed the phosphorylation status of Ulk1 at two different sites (Ser555 and Ser757), which are known to be essential for the coordination of AMPK and mTOR concurrent activity (Russell et al., 2014), and the AMPK-dependent phosphorylation of the Raptor at Ser792 (Gwinn et al., 2008). Analysis of the AMPK-mediated phosphorylation of Ulk1 at Ser555 (Egan et al., 2011; Kim et al., 2011) showed that this phosphorylation decreased in WT fibroblasts, but not in *Col6a1*^-/-^ fibroblasts, after serum withdrawal (**Figure 4D**). In addition, Raptor phosphorylation appeared to be decreased in ColVI null cells after serum depletion, with respect to WT (**Figure 4D**), in agreement with a decreased AMPK-mediated inhibition of mTOR activity. Consistently, mTOR dependent phosphorylation of Ulk1 at Ser757, able to prevent Ulk1 and AMPK interaction and linked to autophagy inhibition (Kim et al., 2011), showed a persistent phosphorylation in *Col6a1*^-/-^ fibroblasts after serum deprivation (**Figure 4D**). A schematic representation of these findings and their impact on autophagy regulation in WT and *Col6a1*^-/-^ fibroblasts is shown in **Figure 4E**.

### Autophagy Is Modulated by Adhesion Onto ColVI

**FIGURE 5:**
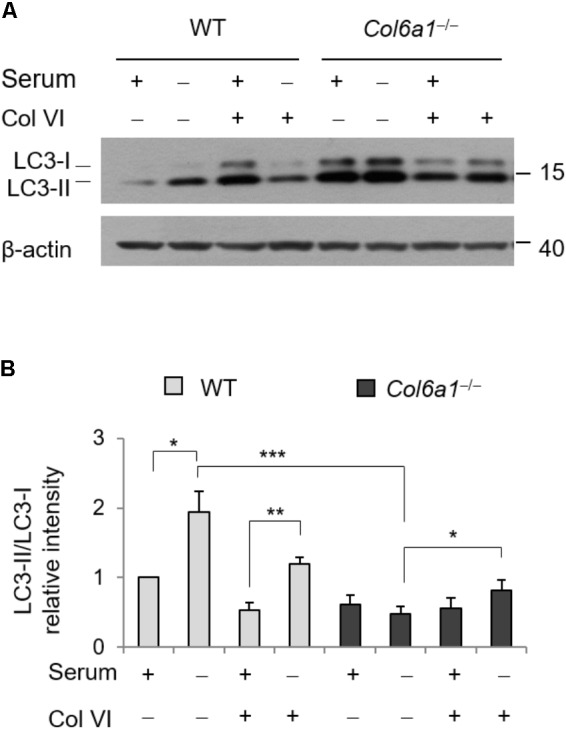
Autophagy is modulated by ColVI. **(A)** Western blotting of LC3 lipidation in total cell extracts from WT and *Col6a1*^-/-^ fibroblasts maintained in complete medium or subjected to serum withdrawal for 3 h. When indicated, cells were grown onto purified ColVI, provided as adhesion substrate. **(B)** Densitometric quantifications of LC3-II/LC3-I ratio, calculated from three independent western blot experiments as shown in **(A)**. For both WT and *Col6a1*^-/-^ fibroblasts, values for cells grown in complete medium in the absence of plated ColVI were arbitrarily set to 1. ^∗^*P* < 0.05, ^∗∗^*P* < 0.01, ^∗∗∗^*P* < 0.001.

To evaluate whether addition of ColVI could impinge on autophagy, we cultured WT and *Col6a1*^-/-^ fibroblasts onto purified ColVI and subjected them to serum starvation as described above. Culture onto native ColVI elicited an effect on LC3 lipidation in both WT and *Col6a1*^-/-^ fibroblasts (**Figure 5**). Indeed, starvation-induced lipidation of LC3 was increased when *Col6a1*^-/-^ fibroblasts were cultured onto ColVI (**Figures 5A,B**). Analysis of the LC3-II/-I ratio showed that basal autophagy in complete medium was decreased in WT fibroblasts adhering on ColVI, but starvation-induced stimulation of autophagy was maintained (**Figures 5A,B**).

### *Col6a1*^-/-^ Fibroblasts Display Fragmented Mitochondria and Mitophagy Defects

**FIGURE 6:**
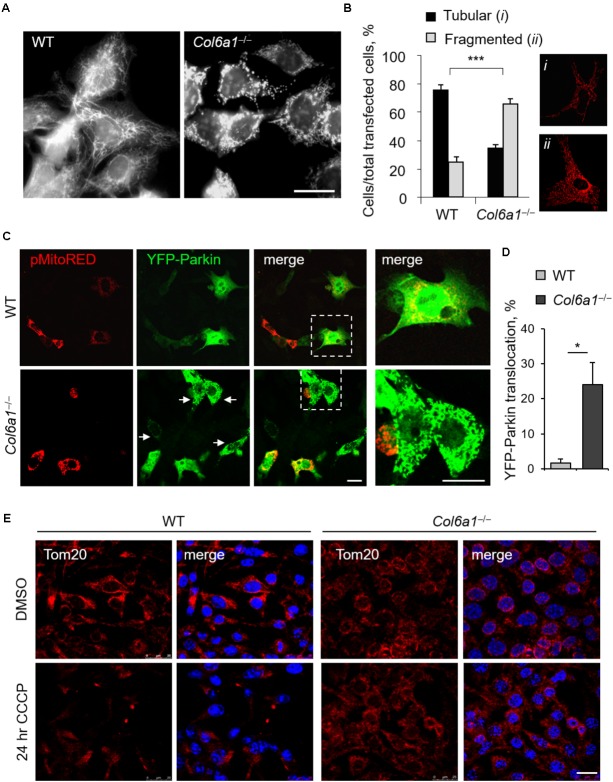
Mitochondrial network is more fragmented and Parkin-dependent mitophagy is impaired in *Col6a1*^-/-^ fibroblasts. **(A)** Representative micrograph of MitoTracker-labeled mitochondria of WT and *Col6a1*^-/-^ fibroblasts. Scale bar, 25 μm. **(B)** Quantification of cells showing tubular (*i*) or fragmented (*ii*) mitochondria network in WT and *Col6a1*^-/-^ fibroblasts, following transfection with mitochondrial fluorescent plasmids (pMitoRed, as in the representative panels *i* and *ii*). Data represents the mean of at least five independent biological replicates. ^∗∗∗^*P* < 0.001. **(C)** Representative micrographs of WT and *Col6a1*^-/-^ fibroblasts transfected with YFP-Parkin and pMitoRed plasmids. Arrows point at mitochondria-translocated YFP-Parkin. The right panels show a higher magnification of the boxed area of the respective merged image. Scale bar, 25 μm. **(D)** Quantification of cells with mitochondria-located YFP-Parkin in WT and *Col6a1*^-/-^ fibroblasts, as in **(C)**. Data represents the mean of at least three independent biological replicates. ^∗^*P* < 0.05. **(E)** Immunostaining for the mitochondrial marker Tom20 (red) in WT and *Col6a1*^-/-^ fibroblasts following 24 h treatment with 20 μM CCCP (or DMSO as a control). Nuclei were stained with Hoechst (blue). Scale bar, 25 μm.

In agreement with previous finding in cell cultures derived from muscle biopsies of UCMD and BM patients (Sabatelli et al., 2012), *Col6a1*^-/-^ fibroblasts displayed a higher percentage of fragmented mitochondria than WT fibroblasts (**Figure 6**). We labeled mitochondria with different probes, using MitoTracker, pMitoRed and Mito-YFP plasmids, and we consistently found that fragmented mitochondria were more than twofold increased in *Col6a1*^-/-^ fibroblasts when compared to WT fibroblasts (**Figures 6A,B**). We then analyzed the efficiency of mitochondria removal via mitophagy, a selective form of autophagy (Youle and Narendra, 2011), by performing co-immunolocalization analysis of mitochondria and autophagosomes/autolysosomes after serum withdrawal. Toward this aim, we used fibroblasts derived from *Col6a1*^+/+^::GFP-LC3 and *Col6a1*^-/-^::GFP-LC3 mice and stained mitochondria and lysosomes with the Tom20 and LAMP-2 markers, respectively. This analysis confirmed defective mitochondria elongation and decreased autophagosome-lysosome fusion in *Col6a1*^-/-^ fibroblasts (**Supplementary Figure S6**). Interestingly, *Col6a1*^-/-^ fibroblasts displayed increased co-localization of mitochondria with autophagosomes/lysosomes when compared to WT fibroblasts (**Supplementary Figure S6B**). To assess whether mitochondrial clearance by autophagy was functional in *Col6a1*^-/-^ fibroblasts, we transfected cells with YFP-Parkin (Narendra et al., 2008) and pMitoRed constructs (**Figure 6C**). Notably, YFP-Parkin was more frequently translocated to mitochondria in *Col6a1*^-/-^ fibroblasts (more than 10-fold increase with respect to WT fibroblasts) already in basal conditions (**Figure 6D**). We evaluated mitochondria removal by monitoring Tom20 immunostaining after 24 hr treatment with CCCP, used as mitochondrial uncoupler, and found that the mitochondrial pool was markedly decreased in WT fibroblasts, but not in *Col6a1*^-/-^ fibroblasts, after CCCP-induced damage (**Figure 6E**).

## Discussion

ColVI exerts a broad range of physiological functions in different tissues, being particularly abundant in skeletal muscle, peripheral nerves and connective tissues (Cescon et al., 2015). Although in skeletal muscle ColVI is produced by fibroblasts, and not by myofibers (Braghetta et al., 2008; Zou et al., 2008), muscles represent the most affected tissue in human pathologies caused by mutations of *COL6* genes, such as BM and UCMD (Bönnemann, 2011; Bushby et al., 2014). Therefore, at difference from other muscular dystrophies and inherited muscle disorders, ColVI-related myopathies represent a unique class of non-cell-autonomous disease of skeletal muscles, since the mutated protein downstream the primary genetic defect is not produced by the myofibers themselves, but by fibroblasts (Zou et al., 2008). Although previous studies revealed that ColVI deficiency in the ECM has a major impact in the survival and homeostasis of muscle fibers (Cescon et al., 2015), the cellular and molecular consequences of ColVI deficiency in fibroblasts themselves are still unknown. In the present study, we investigated the consequences of ColVI ablation in fibroblasts, using immortalized fibroblasts derived from WT and ColVI null mice.

We previously demonstrated that lack of ColVI leads to a failure of the autophagic machinery in skeletal muscles, impinging on myofiber homeostasis and turnover of damaged organelles, and finally causing myofiber death (Irwin et al., 2003; Grumati et al., 2010). Our present data reveal that ColVI ablation also impinges on fibroblast homeostasis, thus suggesting that the muscle damage which arises in *Col6a1*^-/-^ mice may be sustained and exacerbated by fibroblast intrinsic defects and pointing at fibroblasts as active players in the pathogenesis of ColVI-related diseases. Our findings indicate that fibroblasts lacking ColVI are not able to fulfill their autophagic needs, in particular under stress-inducing conditions. Indeed, *Col6a1*^-/-^ fibroblasts display increased LC3 protein levels that do not correlate with autophagy induction, as revealed by the analysis of LC3 protein levels and gene expression. In parallel, *Col6a1*^-/-^ fibroblasts massively accumulate LC3-positive autophagosomes in basal condition, independently of serum starvation or CQ treatment. These findings indicate that *Col6a1*^-/-^ fibroblasts are not able to degrade autophagosomes and their content, accumulating them inside the cells. In addition, both protein and transcript levels for p62 are decreased in *Col6a1*^-/-^ fibroblasts. Literature studies showed that decreased p62 mRNA translation can be linked to reduced phosphorylation of Erk1/2 (Lee et al., 2010; Kim et al., 2014) and/or aberrant activation of TFEB translational program (Settembre et al., 2011), as indeed displayed by *Col6a1*^-/-^ fibroblasts in our study, which show a marked disbalance of the cytosolic and nuclear pools of TFEB, not only after serum withdrawal but also in complete medium. Altogether, these findings strongly support the concept of an impairment of the autophagy-lysosome machinery in ColVI-deficient fibroblasts.

It is well established that proper activation of autophagy is essential for the turnover of aged and dysfunctional cellular organelles, such as mitochondria (Komatsu et al., 2007; Youle and Narendra, 2011; Schneider and Cuervo, 2014). Mitochondria are the main energy producers of the cell, and they are organized in a very dynamic network regulated by fusion and fission processes, which ensure the maintenance of healthy organelles and act in concert with cell decision programs or remodeling (Youle and Narendra, 2011; Pernas and Scorrano, 2016). We investigated the mitochondrial network morphology and found that *Col6a1*^-/-^ fibroblasts display enhanced mitochondrial fragmentation when compared to WT fibroblasts, a feature often linked to mitochondria dysfunction (Jheng et al., 2012; Toyama et al., 2016). Interestingly, it was recently shown that activation of AMPK is sufficient to promote fragmentation of the mitochondrial network, even in the absence of mitochondrial stress (Toyama et al., 2016). Notably, the mitochondrial fragmentation displayed by *Col6a1*^-/-^ fibroblasts resembles the alterations found in primary cell cultures derived from BM and UCMD muscle biopsies (Sabatelli et al., 2012). The enhanced mitochondrial fission in *Col6a1*^-/-^ fibroblasts is also associated with mitochondrial translocation of Parkin, a protein that is actively recruited to the outer mitochondrial membrane of damaged mitochondria (Narendra et al., 2008). Despite the high rate of Parkin translocation in *Col6a1*^-/-^ fibroblasts, mitochondria turnover does not occur properly in these cells, as confirmed by their response to mitochondria-depolarizing treatments, likely due to the inability of *Col6a1*^-/-^ fibroblasts to correctly activate and complete the autophagic process.

To throw light into the defects displayed in *Col6a1*^-/-^ fibroblast, we analyzed the interplay between AMPK, Akt and mTORC1 signaling, which is essential for the autophagic response to nutrients (Russell et al., 2014). AMPK is an energy sensor of the cell, becoming active under metabolic stress (Russell et al., 2014). The markedly increased AMPK phosphorylation we found in *Col6a1*^-/-^ fibroblasts points at a strong energy impairment in these cells, both in basal and under starvation conditions. AMPK is involved in autophagy activation, via Ulk1 phosphorylation and mTORC1 inhibition (Kim et al., 2011; Shang and Wang, 2011; Russell et al., 2014). Indeed, AMPK controls the recruitment of 14-3-3 proteins to mTORC1 complex and its inactivation by phosphorylation of the subunit Raptor at its Ser792 residue (Gwinn et al., 2008). In *Col6a1*^-/-^ fibroblasts, we found that AMPK-dependent phosphorylation of Ulk1 at its specific target residue Ser555 is acting toward autophagy activation when nutrients are depleted. In addition, these cells show a trend toward increased phosphorylation of Raptor. Despite AMPK pathway activation, upon serum starvation *Col6a1*^-/-^ fibroblasts display increased activity of Akt, a serine threonine kinase which negatively regulates autophagy induction in mammalian cells, by acting on mTORC1 signaling (Manning and Cantley, 2007). In agreement with this, we found increased mTORC1 activation in *Col6a1*^-/-^ fibroblasts concurrent with autophagy induction by starvation, as revealed by enhanced phosphorylation of the mTORC1 downstream targets rpS6 and 4E-BP1. The increased mTORC1 activity further elicits an increase in the inhibitory Ser757 phosphorylation of Ulk1, which is critical in antagonizing the interaction between Ulk1 and AMPK and inhibiting autophagy induction (Egan et al., 2011; Kim et al., 2011). Thus, although AMPK over-activation is expected to enhance autophagy induction in *Col6a1*^-/-^ fibroblasts, the process is strongly counteracted by active mTORC1, leading to autophagosome accumulation and an impairment of the autophagic flux in *Col6a1*^-/-^ fibroblasts (Castagnaro, 2015).

The accumulation of autophagic vesicles we observed in ColVI-deficient fibroblasts may also rely on impaired lysosomal degradation (Huynh et al., 2007). The presence of lysosomal defects in *Col6a1*^-/-^ fibroblasts under basal condition, as revealed by the presence of enlarged lysosomes together with lower LAMP-2 levels, could be sufficient to impair autophagosome-lysosome fusion. Indeed, LAMP-1 and LAMP-2 are required for proper autolysosome formation and hence for the removal of autophagosomes (Huynh et al., 2007). In addition, it is known that during autophagy the lysosomal function itself is regulated by the fusion of autophagosomes with lysosomes and relies upon mTORC1 suppression (Zhou et al., 2013). Thus, the alteration of lysosomal morphology in *Col6a1*^-/-^ fibroblasts is consistent with the sustained mTORC1 activity and the decreased autolysosome formation. Taken together, these results point at a failure of autophagy completion in *Col6a1*^-/-^ fibroblasts, sustained both by defective lysosomal function and by mTORC1 deregulation. In this context, it is worthy to underline that also the regulation of TFEB, a master regulator of lysosomal biogenesis, activated in the presence of lysosomal stress (Settembre et al., 2011), is markedly altered in the absence of ColVI.

Although deeper investigations are needed to better clarify the effects of fibroblast adhesion onto ColVI, our findings suggest that ColVI *per se* owns prosurvival effects and autophagy-instructive properties, similarly to what was already seen for other ECM proteins (Tuloup-Minguez et al., 2011; Neill et al., 2014). Our work opens the possibility for future studies aimed at dissecting the molecular signals involved in this regulation and in the transduction of autophagy regulatory effects from the extracellular milieu within the cells. Indeed, several ECM proteins, including decorin, laminin-2, kringle V, endostatin, and endorepellin, are emerging as primary regulators of autophagy (Neill et al., 2014, 2017). Among collagens, collagen types I and IV were found to elicit autophagy modulation in HeLa cells when provided as adhesion substrates, but a detailed mechanistic understanding of these effects is still lacking (Tuloup-Minguez et al., 2011). In addition, ECM modulation of autophagy often takes place in a nutrient availability-independent manner, suggesting an intrinsic ability to supervise and constrain extracellular factors that trigger this catabolic response, in order to harmonize cell responses (Neill et al., 2014).

Future work is needed to understand in detail the intricate and multifaceted aspects of ColVI biology and functions in skeletal muscles and in other tissues. On the other hand, the present study provides further insights into the dynamic nature of the ECM, in particular of one of the major constituents of muscle ECM. ColVI produced by fibroblasts in the muscle-associated connective tissue may allow the fine-tuning of skeletal muscle response to different types of stress, as underlined by the fact that ColVI deficiency causes muscle pathology in mice and humans. In addition, the findings of the present study provide novel clues for the pathomolecular defects of ColVI-related diseases and for the identification of new targets for therapy. Indeed, it is well known that fibroblasts and connective tissue actively cooperate with myofibers and other cell types to maintain the physiological properties and activities of skeletal muscle (Murphy et al., 2011). Finally, considering the increasing need for non-invasive strategies in clinical studies of ColVI-related myopathies (Castagnaro et al., 2016), these findings pave the way for the prospective use of fibroblasts in clinical studies aimed at monitoring the response to therapeutic treatments in ColVI-related disorders.

## Author Contributions

SC designed and performed the experiments, analyzed, provided interpretation to the data, and wrote the manuscript. MCh performed quantitative RT-PCR and contributed to manuscript writing and revision. MCe assisted with mitochondrial measurements. PBr contributed to manuscript writing. PG conceived and designed the experiments. PBo coordinated the study, and contributed to manuscript writing and revision.

## Conflict of Interest Statement

The authors declare that the research was conducted in the absence of any commercial or financial relationships that could be construed as a potential conflict of interest.

## Abbreviations

3-MA: 3-methyladenine
AMPK: AMP-activated protein kinase
BM: Bethlem myopathy
CCCP: carbonyl cyanide m-chlorophenylhydrazone
ColVI: collagen VI
CQ: chloroquine
ECM: extracellular matrix
Erk1/2: extracellular signal-regulated kinases 1 and 2
LAMP-2: lysosomal-associated membrane protein 2
MAP1LC3B/LC3: microtubule-associated protein 1 light chain 3B
MEF: mouse embryonic fibroblasts
mTOR: mammalian target of rapamycin
PI: propidium iodide
Raptor: regulatory-associated protein of TOR
rpS6: ribosomal protein S6
SQSTM1/p62: sequestosome 1
TFEB: transcription factor EB
UCMD: Ullrich congenital muscular dystrophy
Ulk1: Unc-51 like autophagy activating kinase 1
