# Supplementary material for: Extracellular Collagen VI Has Prosurvival and Autophagy Instructive Properties in Mouse Fibroblasts
Source: Front Physiol. 2018 Aug 17;9:1129. doi: 10.3389/fphys.2018.01129 (PMC6107713; doi:10.3389/fphys.2018.01129)
Supplement: Supplementary file 1 [file Presentation_1.pdf]

Supplementary Information

Extracellular collagen VI has prosurvival and autophagy instructive properties in mouse embryonic fibroblasts

Silvia Castagnaro, Martina Chrisam, Matilde Cescon, Paola Braghetta, Paolo Grumati, Paolo Bonaldo

Supplementary Figures S1-S6

Supplementary Table S1, S2

Supplementary Figure S1

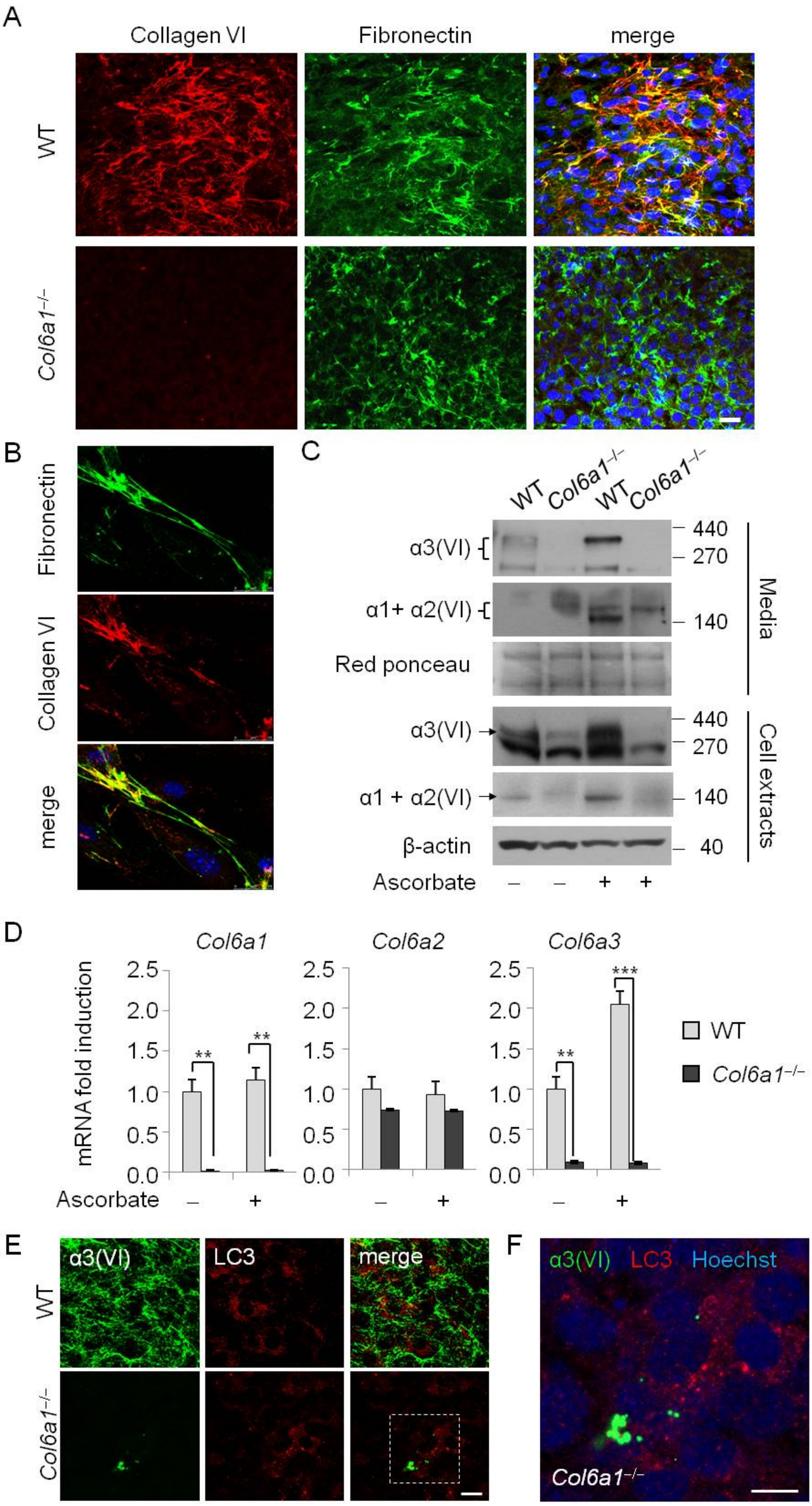

Supplementary Figure S1. Characterization of ECM deposition and collagen production by immortalized fibroblast cultures derived from wild-type and *Col6a1*$^{-/-}$ mice. (**A**) Immunofluorescence for ColVI (red) and fibronectin (green) in WT and *Col6a1*$^{-/-}$ fibroblast cultures. Scale bar, 25 µm. (**B**) Confocal microscopy images of ColVI (red) and fibronectin (green) co-immunostaining in WT fibroblasts. Nuclei were stained with Hoechst (blue). Scale bar, 25 µm. (**C**) Western blot analysis for ColVI chains in culture media and cell extracts from WT and *Col6a1*$^{-/-}$ fibroblasts. Where indicated, 0.25 mM ascorbic acid was used to induce ColVI secretion. (**D**) qRT-PCR analysis of *Col6a1*, *Col6a2* and *Col6a3* mRNA levels in WT and *Col6a1*$^{-/-}$ fibroblasts. Data represent the mean of at least three independent experiments. **, $P < 0.01$; ***, $P < 0.001$. (**E, F**) Co-immunofluorescence staining for α3(VI) chain (green) and LC3 (red) in confluent WT and *Col6a1*$^{-/-}$ fibroblasts cultures. A higher magnification of the boxed area is shown in panel **F**. Intracellular dots of α3(VI) are detected in *Col6a1*$^{-/-}$ fibroblasts, with no co-localization with autophagosomes. Nuclei were stained with Hoechst (blue). Scale bars, 20 µm (**E**) or 10 µm (**F**).

Supplementary Figure S2

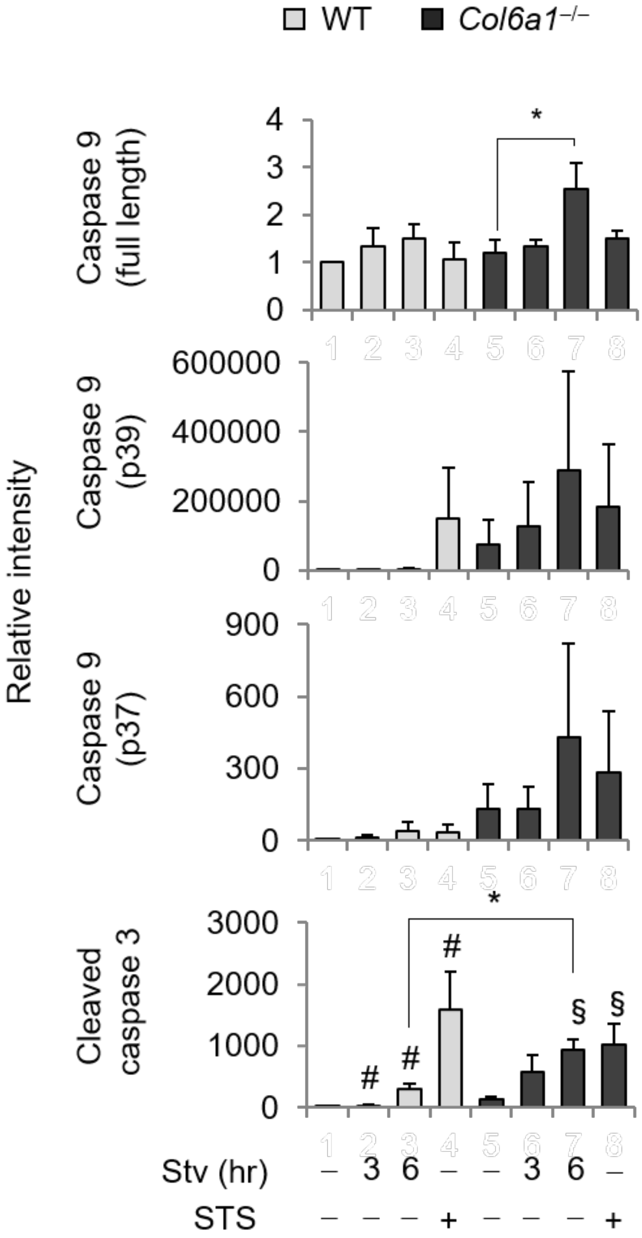

Supplementary Figure S2. Densitometric quantification of caspases 9 and 3 in WT and *Col6a1*$^{-/-}$ fibroblasts in basal, serum-depleted, and staurosporine treated condition. Histogram showing the relative intensity (on the loading control) of full length caspase 9, p37 and p39 cleaved caspase 9, and cleaved caspase 3, as determined by three independent western blots of total cell extracts from WT and *Col6a1*$^{-/-}$ fibroblasts cultured in complete DMEM with 10% serum, or following serum starvation for 3 hr and 6 hr, as in Fig. 1B. *, $P < 0.05$; #, $P < 0.05$ for the comparison between WT complete medium and WT no serum; §, $P < 0.05$ for the comparison between *Col6a1*$^{-/-}$ complete medium and *Col6a1*$^{-/-}$ no serum. STS, staurosporine; Stv, serum starvation.

Supplementary Figure S3

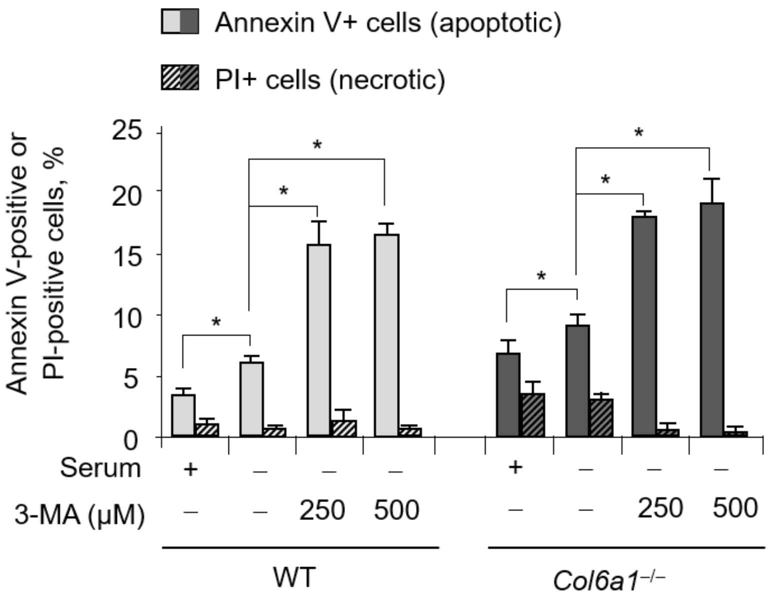

Supplementary Figure S3. The autophagy inhibitor 3-MA increases apoptosis in a dose-dependent manner in both WT and *Col6a1*$^{-/-}$ fibroblasts. Quantification of annexin V-positive (apoptotic) and PI-positive (necrotic) cells, as determined by flow cytometry analysis of annexin V and PI in WT and *Col6a1*$^{-/-}$ fibroblasts cultured for two days in complete medium (Serum+) or following serum starvation conditions for 3 hr (Serum–), in the absence or presence of 3-MA at the indicated concentrations. Data represent the mean of at least three independent experiments. *, $P < 0.05$.

Supplementary Figure S4

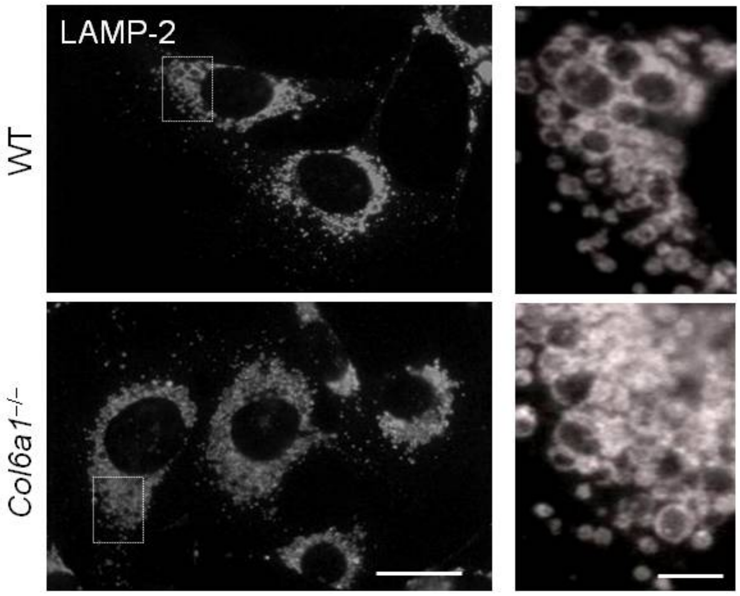

Supplementary Figure S4. CQ treatment induces lysosome swelling in fibroblasts. Immunofluorescence for LAMP-2, showing similar immunostaining in WT and *Col6a1*$^{-/-}$ fibroblasts after 50 μM CQ treatment. The right panels show a higher magnification of the boxed area of the respective left panels, with a detailed view of perinuclear swollen lysosomes. Scale bars, 25 μm (left panels) and 5 μm (right panels).

Supplementary Figure S5

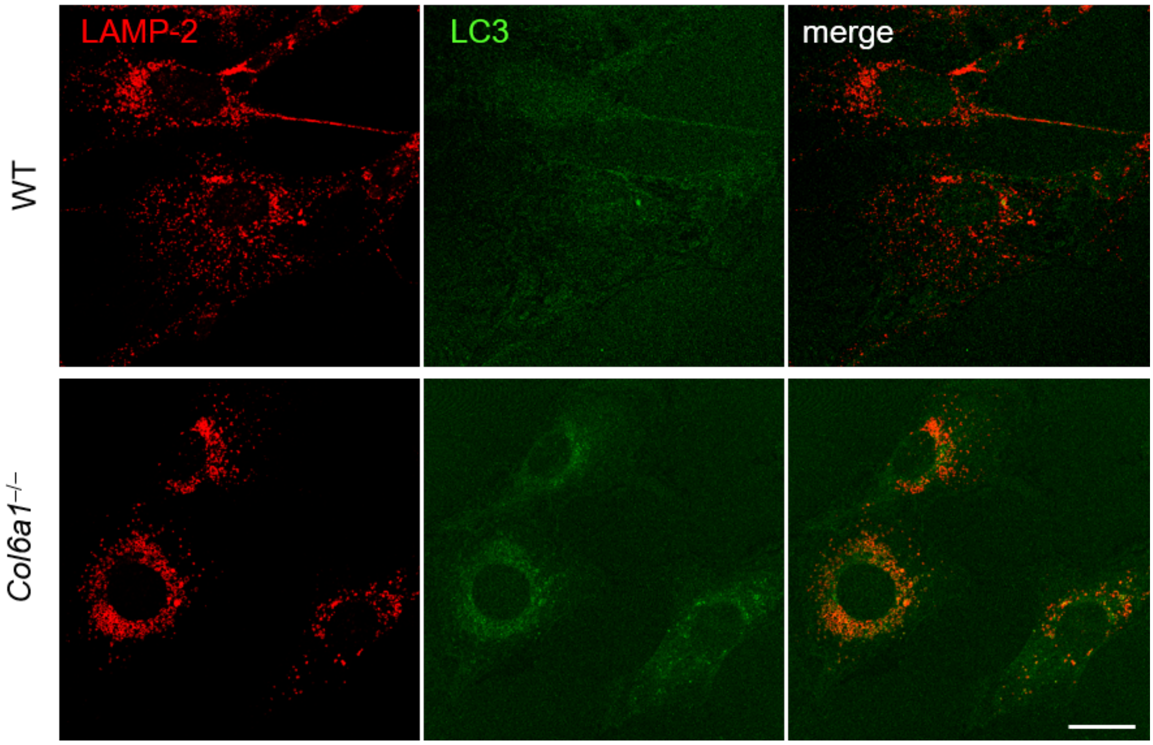

Supplementary Figure S5. Co-localization analysis of autophagosome and lysosomes in WT and *Col6a1*$^{-/-}$ fibroblasts. Co-immunostaining for LC3 (autophagosome marker) and LAMP-2 (lysosome marker) in WT and *Col6a1*$^{-/-}$ fibroblasts cultured in complete medium. Scale bar, 25 µm.

Supplementary Figure S6

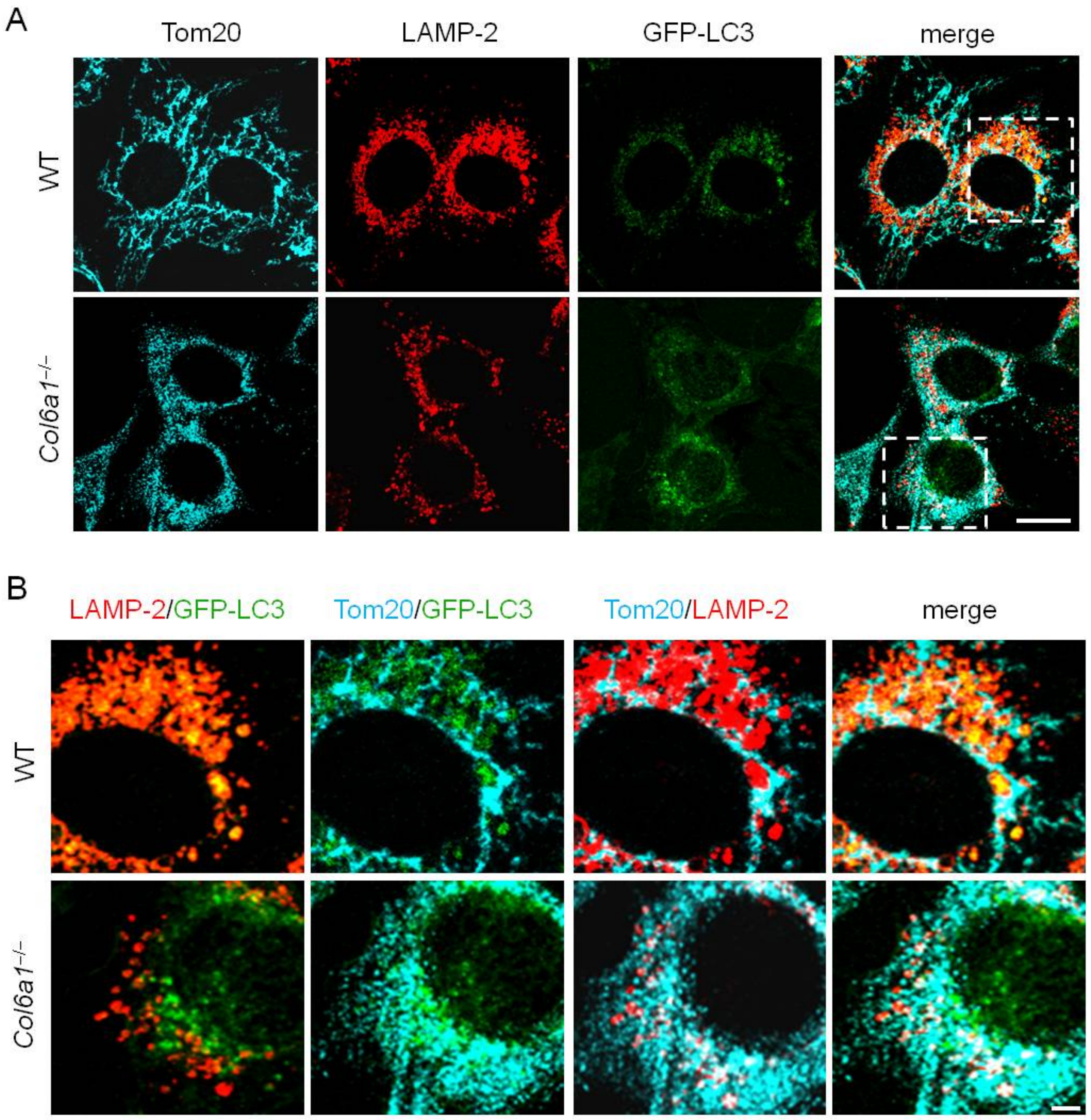

Supplementary Figure S6. Co-localization analysis of mitochondria, autophagosomes and lysosomes in WT and *Col6a1*$^{-/-}$ fibroblasts. (**A**) Immunofluorescence for Tom20 (mitochondria, cyan) and LAMP-2 (lysosomes, red) in *Col6a1*$^{+/+}$::GFP-LC3 (WT) and *Col6a1*$^{-/-}$::GFP-LC3 (*Col6a1*$^{-/-}$) fibroblasts cultured for two days and subjected to serum starvation for 3 hr. Scale bar, 25 µm. (**B**) Higher magnification details of the dotted areas shown in the merge images of panel **A**. Color codes are indicated on the top. *Col6a1*$^{-/-}$ fibroblasts display increased co-localization of fragmented mitochondria (cyan) with autophagosomes (green) and lysosomes (red). Scale bar, 25 µm.

Supplementary Table S1. Primers used for the qRT-PCR.

	**Forward primer (5' > 3')**	**Reverse primer (5' > 3')**
Col6a1	TGCCCTGTGGATCTATTCTTCG	CTGTCTCTCAGGTTGTCAATG
Col6a2	CAACCGCATCATCAAGGTCA	GGGTCTCCCTGTCGTCCTTT
Col6a3	AACCCTCCACATACTGCTAATTC	TCGTTGTCACTGGCTTCATT
Map1lc3b	CACTGCTCTGTCTTGTGTAGGTTG	TCGTTGTGCCTTTATTAGTGCATC
Sqstm1	CCCAGTGTCTTGGCATTCTT	AGGGAAAGCAGAGGAAGCTC
Becn1	TGAATGAGGATGACAGTGAGCA	CACCTGGTTCTCCACACTCTTG
Bnip3	TTCCACTAGCACCTTCTGATGA	GAACACCGCATTTACAGAACAA
Lamp2	CAAAAGGACAGTATTCTACAGCTCA	CCACCGCTATGGGCACAA
Tfeb	TCAGAAGCGAGAGCTAACAGAT	TGTGATTGTCTTTCTTCTGCCG
Lamp1	AGTGGGAGTTGCGGTATCAAC	TGGAGATGCTGAATGTGGGC
Atg14	GCAGGTCAGGACCCTTTGAA	TCCTCATCGCTTACACGCTC
Uvrag	CATCGCTGCTCGGAACATTG	CCTCCACGTCGGATTCAAGG
Gapdh	CACCATCTTCCAGGAGCGAG	CCTTCTCCATGGTGGTGAAGAC

Supplementary Table S2. Antibody list.

Antibody	Working dilution	Source
Akt	1:1000	9272, Cell Signaling Technologies
phospho-Akt	1:1000	4058, 4060, Cell Signaling Technologies
AMPK	1:1000	2532, Cell Signaling Technologies
phospho-AMPK	1:1000	4188, Cell Signaling Technologies
β-actin	1:5000	A 5316, Sigma-Aldrich
Caspase 3	1:1000	9661, Cell Signaling Technologies
Caspase 9	1:1000	9504, Cell Signaling Technologies
Collagen VI, α1(VI)	1:300 (IF); 1:1000 (WB)	H-200, sc-20649, Santa Cruz Biotechnology
Erk1/2	1:1000	9102, Cell Signaling Technologies
phospho-Erk1/2	1:1000	4377, Cell Signaling Technologies
LAMP-2	1:200 (IF); 1:500 (WB)	GL2A7, ab13524 Abcam
LC3B	1:1000 (WB)	PA1-16930, Thermo Scientific
LC3B	1:200 (IF); 1:1000 (WB)	2775, Cell Signaling Technologies
p62	1:5000 (WB)	GP62-C, Progen
p62	1:5000 (WB)	PM045, MBL
rpS6	1:1000	2217, Cell Signaling Technologies
phospho-rpS6	1:1000	5364, Cell Signaling Technologies
Tom20	1:200	sc-11415, Santa Cruz Biotechnology
4E-BP1	1:1000	9452, Cell Signaling Technologies
phospho-4E-BP1	1:1000	2855, Cell Signaling Technologies
Ulk1	1:1000	4773, Cell Signaling Technologies
phospho-Ulk1 (Ser555)	1:1000	5869, Cell Signaling Technologies
phospho-Ulk1 (Ser757)	1:1000	6888, Cell Signaling Technologies
Raptor	1:1000	2280, Cell Signaling Technologies
phospho-Raptor	1:1000	2083, Cell Signaling Technologies

IF, immunofluorescence; WB, western blot.
